# Dr. Maurice B. Perkins

**Published:** 1901-07

**Authors:** 


Dr. Maurice B. Perkins, of Schenectady, professor of chemistry at Union College and at Albany Medical College, died suddenly June 18, 1901, aged 65 years. Dr. Perkins served for many years as a member of the state board of health. He also took prominent part in the proceedings of the Medical Society of the State of New York for a number of years and thus became well known to the general profession of medicine. His loss will be deeply felt by the colleges in which he taught as well as by his multitude of friends throughout the country.

